# Attitudes to Brain Health and Dementia Amongst the Chinese Population Living in the UK

**DOI:** 10.1177/14713012251356529

**Published:** 2025-06-29

**Authors:** Richard Cheston, Mei Champ, Jennifer N. W. Lim

**Affiliations:** 1 Professor of Dementia Research, School of Social Sciences, 1981 University of the West of England, Bristol, UK; 2 Senior Lecturer and Interim Deputy Dean (Teaching, Learning and Assessment), 1981 University of the West of England, Bristol, UK; 3 Reader in Health Inequalities and Behavioural Science, School of Health and Society, 8695 University of Wolverhampton, UK

**Keywords:** dementia attitudes monitor, Alzheimer’s disease, stigma, risk, awareness, prevention

## Abstract

**Background:** Just under half a million people who identify as Chinese or Chinese-British are living in the UK. Chinese migrants have a distinctive linguistic and cultural heritage and are likely to differ from the wider UK population in their attitudes to dementia care. However, to date no studies have explored this area. This study aimed to compare attitudes to dementia amongst Chinese people and the wider UK population using a translated version of the 2023 Dementia Attitudes Monitor survey (DAM). **Methods:** We translated the DAM into Simplified and Traditional Chinese and distributed this through an online survey. In total 84 UK based participants (65 women and 19 men) completed the survey. We weighted data by age and sex. **Results:** We identified important differences between Chinese participants and the wider UK population. Chinese participants were more likely to report that they would find it hard to talk to someone with dementia and that they would not feel comfortable telling people outside their close family if they were to be diagnosed. Higher levels of knowledge about dementia were associated with increased reluctance to tell people outside their family. Chinese participants were equally willing to take a test that could tell them whether they were in the early stages of dementia, even before symptoms showed. They were also more likely to report that they would want to know information in midlife about their risk of developing dementia later on. **Conclusion:** This paper is the first to report knowledge about brain functioning and dementia within the UK-based Chinese community. Chinese people are highly motivated to reduce their dementia risk – but to do so requires specific public health programmes that are adapted to meet the specific needs of Chinese communities.

## Introduction

According to the 2021 UK census just under half a million people are living in the UK who identify as Chinese or Chinese-British, which amounts to 0.7% of the overall UK population. While Chinese people have migrated to the UK from many different countries, including Malaysia, Singapore, and Vietnam as well as from mainland China, the oldest and largest grouping are of Hong Kong Chinese. This movement of people intensified after January 2021 when the British Nationals (Overseas) visa scheme was introduced (Rolfe & Benson, 2023). There are now over 120,000 people from Hong Kong living in the UK, and this number is set to increase dramatically with many estimates suggesting that another 300,000 Hong Kongers may migrate to the UK in the next few years (Rolfe & Benson, 2023).

The Chinese population in the UK reflects the diversity found across Chinese cultures globally and includes a wide range of political, cultural and social backgrounds. Similarly, Chinese people living in the UK often use different spoken and written languages, which may reflect subtly different social attitudes. For instance, someone who grew up in Hong Kong might speak both Cantonese and Hakka but is unlikely to speak Mandarin. In contrast, many people from mainland China typically speak Mandarin. There is some evidence of a difference in preferred terms for dementia between Mandarin and Cantonese speakers with the former showing a slight preference for *chi dai zheng*,^1^ the standard medical term used in clinical and academic contexts to describe dementia (Champ et al., forthcoming). Importantly, this term carries a number of negative connotations as *chi* can mean crazy, and *dai* can be interpreted as catatonic or foolish. Concern has therefore been expressed that the stigma associated with the term *chi dai zheng* discourages open discussions and seeking professional help (Asada et al., 2022; Fedotova et al., 2023). As a consequence, alternative terms for dementia have begun to be used including the term *tui zhi zheng*, which translates into English as a reduced mental capacity, or *nao tui hua zheng*, meaning degenerative brain disorder.

### Chinese People and Dementia

There is a growing body of research about the dementia knowledge and attitudes of people from the global Chinese diaspora which suggest that there are differences in knowledge about dementia and attitudes towards this condition (Yang et al., 2015; Zhang et al., 2023). Thus, in the US, Chinese American women caring for relatives with dementia were more likely to believe that Alzheimer’s disease was a normal part of ageing than were white caregivers (Gray et al., 2009), while Asian older adults had less knowledge about Alzheimer’s disease than did Anglo older adults (Ayalon & Arean, 2004). There may also be attitudinal differences between Chinese migrants and those still living in China. Thus, Zhao et al. (2022) found that Chinese migrants in Melbourne were more avoidant of dementia and people living with dementia and were more likely to perceive a need for dementia-related educational activities than were participants living in Beijing.

While this research suggests that it is likely that Chinese people living in the UK, with their distinctive linguistic and cultural heritage, will differ both from the wider UK population in terms of their attitudes both to dementia and to the provision of care, and potentially from people living in China, relatively few studies have, to date, explored this area. Bifarin et al. (2022) looked at the attitudes to care of UK-based Chinese students with families in China to the prospect of caring for their parents when they were older within the context of intergenerational ties and filial obligations (Xiao or 孝). Adopting a social constructivist position, they conducted three focus groups with 19 students, using a semi-structured topic guide which suggested a strong ‘*culture of duty*’ within which familial obligation and societal expectations were prominent. There is also some evidence that attitudes towards dementia may differ significantly amongst people from Chinese communities living in the UK compared to the wider, UK population (Yang et al., 2015; Zhang et al., 2023). Thus, within Confucian beliefs, illnesses such as dementia may be viewed as arising from a lack of harmony within the individual and their family (Sun et al., 2012). This imbalance might arise, for instance, from previous acts or behaviour (All-Party Parliamentary Group APPG on Dementia, 2013) such as family disagreements (Cheston et al., 2017). Moreover, as talking about dementia risks exposing a part of oneself, and one’s family to outside judgement and thus creates distress and anxiety, some Chinese people may be reluctant to discuss it openly (Li et al., 2024). Thus, speaking through an interpreter, one Chinese woman reported by Baghirathan et al. (2020), described how her sons preferred that she should not talk about her husband’s diagnosis of dementia outside the family ‘*because it was not appropriate and […] people might look down on them*’. As a consequence of fears about outside judgement, some studies suggest that dementia carries a high level of stigma within Chinese communities (Wong et al., 2024; Xue et al., 2023) with many people being reluctant to tell even members of their own family and preferring to avoid social forms of care such as support groups or day centres (Cheston et al., 2017).

Given that it is likely that roughly 4,000 Chinese people living in the UK have developed dementia^2^ and that this figure is expected to rise to over 20,000 by 2051, it is important to understand more about attitudes to dementia within this community. However, the little research that has to date been carried out is limited and tends to be gathered in just one city (Baghirathan et al., for instance, recruited only from Bristol) rather than nationally. Moreover, while the qualitative nature of this work generates insights about the experiences of caring, it tells us relatively little about the contrast between Chinese attitudes to dementia and brain functioning compared to the wider population.

### The Dementia Attitudes Monitor Survey

One of the most detailed accounts of dementia attitudes across the United Kingdom (UK) is provided by the Dementia Attitudes Monitor (Alzheimer’s Research UK, 2018, 2021, 2023). These reports consist of data collected from three surveys carried out by Alzheimer’s Research UK (ARUK). Each of these three surveys (also known as waves) sampled attitudes and awareness of dementia across the UK public. The second survey (carried out in 2021) reported on a nationally representative sample of 2,259 adults in the UK aged 18 and over of whom 387 people were from ethnic minorities whilst the third (carried out in 2023) included 338 adults from an ethnic minority background. Both surveys grouped respondents into the following categories: white (1,856 in the second wave and 2,161 in the third wave); Asian/Asian British (127, 128), Black African/Caribbean/Black British (138, 120), Mixed/Multiple ethnic groups (93, 59), Arab (4, 7) and Other (25, 24). Neither of these two surveys separately detailed attitudes from Chinese people living in the UK.

This study had two aims: first to compare attitudes to dementia amongst Chinese people living in the UK with the majority population using a translated version of the survey used in the second and third Dementia Attitudes Monitor reports; and secondly to explore whether greater knowledge about dementia was associated with differences in attitudes.

## Methodology

### Design

Ethical approval was obtained from the Faculty of Education, Health and Wellbeing, University of Wolverhampton.^3^ With the permission of Alzheimer’s Research UK, a member of the research team MC (who is a chartered linguist) translated questions from the ARUK’s Dementia Attitude Monitor (DAM) and Think Brain Health (TBH) Quiz into both Simplified and Traditional Chinese. The translation was then independently moderated in two different cities (Manchester and Bristol) to allow for further refinement.

### Measures

#### Demographic Questions

We asked participants to identify their age, sex, country of origin, country of residence, preferred language, age at leaving full-time education, occupation and whether they knew anyone who had been diagnosed as having dementia. As many respondents completed their education outside the UK, we used the following equivalents to the categories used in all three DAM surveys to identify levels of education: primary school, form 5 (for someone from Hong Kong), year 7 and sixth grade were all as categorised leaving without qualifications; middle or secondary school were coded as a similar level to GCSE/O levels/NVQs; and junior high school, high school or college were treated as equivalent to A levels. We used the NRS Social Grade classification to assign an approximate social grade to participants based on their stated occupation.^4^ However, caution must be taken in interpreting this data as we only asked participants to identify their occupation and not the occupation of the Chief Income earner in the household, and we did not ask pensioners to identify if they received an occupational pension in addition to any state pension. Accordingly, we have categorised people who self-identified as ‘housewife’ and ‘student’ separately.

#### Dementia Attitude Monitor (DAM)

We focused our questions on three areas: awareness and understanding; stigma; and diagnosis and risk. The final Simplified and Traditional Chinese translations, together with the original English version, were then incorporated into an online survey. As the 2021 and 2023 DAM surveys were both conducted via telephone interviews, we modified some questions to enable them to be included in an online survey. Thus, the question “*What, if anything, do you think could increase a person’s risk of developing dementia?*” was originally asked as an open-ended question, allowing respondents to answer spontaneously. Instead, in our online version we asked participants to choose from a list of potential responses.

*The Think Brain Health quiz* consisted of eight statements or questions about brain functioning and dementia which respondents scored as being either correct or incorrect (see Appendix One). On responding, the correct answer was revealed with a short explanatory text. We divided participants into two groups using the median score of 6 out of 8 as a cut off with participants who scored below this being treated as low in knowledge of brain functioning and participants with scores of 6 and above as having a high level of knowledge.

### Distribution

This survey was part of a larger project which was supported by Chinese-led community and voluntary organisations in the five UK cities with the highest Chinese population (London, Manchester, Birmingham, Liverpool and Bristol). These organisations advertised the survey to their members, and we also distributed a link to the survey through social media as well as contacting other networks and Chinese organisations that might be able to help distribute the survey. One of these organisations (the Manchester Chinese Health Information Centre) and an additional organisation (the Chinese in Wales Association based in Swansea) helped their members to complete the survey which was open between August and December 2022.

### Participants

In total, 102 people completed our survey. We excluded from analysis participants who stated that their country of residence was Hong Kong (12 people), China (3), USA (1), Taiwan (1) and Belgium (1). Of the remaining 84 UK based participants, 65 were women with a mean average age of 52.1 years of age (SD = 15.43) and 19 men whose mean age was 27.1 (SD = 15.46). Details of the demographic profile of participants are given in Table 1.

**Table 1.** Demographic Characteristics of Participants

	Female (*n* = 65)	Male (*n* = 19)	Total (*n* = 84)
Country of origin			
China	35	9	44
Hong Kong	18	5	23
Malaysia	6	2	8
UK	4	3	7
Vietnam	1	0	1
Taiwan	1	0	1
Preferred language			
Cantonese	31	9	40
Mandarin	29	5	34
English	3	3	6
Hakka	1	0	1
English and Cantonese	1	0	1
Welsh	0	1	1
Cantonese and Mandarin	0	1	1
Educational level			
No formal qualifications	4	1	5
Left education at 16 (middle school, secondary school, GCSEs or equivalent)	12	4	16
Left education at 18 (high school, sixth form, a level and college or equivalent)	19	3	22
Undergraduate level (diploma or equivalent)	22	9	31
Postgraduate level (masters, PhD)	8	2	10
Approximate social grade (based on stated occupation)			
A: Higher managerial, administrative or professional	2	0	2
B: Intermediate managerial, administrative or professional	11	5	16
C1: Supervisory or clerical and junior managerial roles, administrative or professional	11	3	14
C2: Skilled manual worker	6	1	7
E: Retired	18	8	26
Housewife	13	0	13
Student	3	1	4
Missing	1	1	1

### Analysis

In order to make our data more representative of the Chinese population in the UK, we used data from the 2021 UK census for people who identified as Chinese to weight our data by age and sex. We also collapsed the four categories (‘*Strongly disagree’,* ‘*Somewhat disagree*’, ‘*Tend to Agree*’ and ‘*Strongly Agree*’) into two categories (‘*Disagree’* and ‘*Agree’*), excluding responses of ‘*Neither Agree nor Disagree*’ from the analysis. We then conducted two different sets of analysis. First, we compared data from our survey with data from the third DAM report using appropriate non-parametric analyses. Secondly, we compared responses in terms of participants’ level of knowledge about brain functioning and dementia using their scores on the Think Brain Health quiz. We adjusted the significance level for each of the three areas that we examined to account for multiple testing.

## Results

### Demographic Characteristics

Chinese participants were significantly more likely to report that they either had dementia themselves, or that a member of their family, a close friend or someone else that they know has been diagnosed as having a form of dementia than were participants in the 2023 DAM survey (*χ*^2^ (1, N = 200) = 4.73, *p* = .030).

### Awareness and Understanding

We conducted a series of 2 × 2 chi-squared analyses using Yates’ correction where appropriate to compare first, of all, the results from our survey with data from the 2023 DAM report, and, secondly, to explore whether levels of knowledge about brain functioning and dementia were associated with responses amongst Chinese participants. The differences in agreement levels for the statements *“Dementia affects mental aspects of a person’s health”* and *“Dementia affects the physical aspects of a person’s health”* were not significant.

#### Recognition that Dementia is not Inevitable

There was a significant difference at the adjusted significance level of .017 between the two populations in their levels of agreement with the statement *“Dementia is an inevitable part of getting older”* (*χ*^2^ (1, N = 159) = 12.92, *p* = .0003) with the Chinese population more likely to disagree than to agree. Greater knowledge of brain functioning and dementia was also associated with being more likely to agree with this statement (*χ*^2^ (1, N = 64) = 2.17, *p* = .009).

#### Dementia as a Cause of Death

Comparing our survey with the 2023 DAM study, there was a significant difference in whether participants agreed or disagreed that dementia was a cause of death (*χ*^2^ (1, N = 153) = 13.96, *p* = .0002) with the Chinese population more likely to disagree than to agree with the statement *“Dementia is a cause of death”* compared to the wider UK population. Comparing those with more or less knowledge of dementia within our survey using a chi-squared test with Yates’ correction also showed a difference (*χ*^2^ (1, N = 58) = 6.87, *p* = .009) with people with more knowledge being more likely to agree with the statement *“Dementia is a cause of death”* than to disagree.

We ranked participants’ responses to the question *“What words come to mind when I say dementia?”* and compared the results from our study with those from the most recent DAM report (see Table 2). Using a Mann Whitney U test, the results indicated that there was a difference between the two populations (*z* = 2.30, *p* = .021) with Chinese participants being more likely to identify confusion and a personal connection than were DAM respondents. However, caution must be taken in interpreting this result for two reasons. First, the two procedures used different methods to collect these results (the DAM used an open question, whereas we provided these responses to participants and asked them whether they did or did not agree), and second, with fewer than ten data points, the Mann Whitney test’s approximation to the form of the normal distribution becomes less robust.

**Table 2.** Comparison Between Responses to the Question “*What Words Come to Mind When I Say Alzheimer’s disease*”?

	Chinese UK (*n* = 84)		Dementia attitudes monitor, 3^rd^ wave (*n* = 2,259)	
	Percentage of responses	Rank order	Percentage of responses	Rank order
Memory loss	95	1^st^	52	1^st^
Old age	59	2^nd^=	22	2^nd^
Confusion	59	2^nd^=	11	5^th^
A personal connection	26	4^th^	5	7^th^
An emotional response	25	5^th^	17	3^rd^
Physical changes	23	6^th^	10	6^th^
General changes	15	7^th^	14	4^th^

### Stigma

#### Engaging With People With Dementia

Using a modified significance level of .017, people from Chinese communities were significantly more likely to agree with the statement *“I would find it hard to talk to someone who has dementia”* (χ^2^ (1, N = 139) = 22.76, *p* < .00001) than were DAM participants. Knowledge of dementia did not affect agreement levels with this statement (*χ*^2^ (1, N = 44) = .70, *p* = .402).

#### Being Personally Affected by Dementia

There was no difference between Chinese people and the wider UK public in the extent to which they agreed or disagreed with the statement *“Dementia is the health condition I fear most about getting in the future”* (χ^2^ (1, N = 153) = 3.12, *p* < .077). Level of Knowledge of dementia did not affect agreement levels with this statement (*χ*^2^ (1, N = 63) = 1.33, *p* = .249).

#### Telling Others

Using a modified significance level of .017 to account for multiple tests, people from Chinese communities were significantly more likely to disagree with the statement *“If I was diagnosed with dementia, I would feel comfortable telling people outside my close family*” (χ^2^ (1, N = 173) = 12.32, *p* = .0005). People with more knowledge of dementia were more likely to disagree with this statement (*χ*^2^ (1, N = 74) = 21.96, *p* < .00001).

### Diagnosis and Risk

#### Preventable Health Conditions

Participants were asked to identify which, if any, of five different health conditions (diabetes, stroke, dementia, heart disease and cancer) were possible for people to reduce their risk of developing. Using a Mann Whitney U test, the results indicated that there was no difference between the two populations (*U* = 12, *z* = 0, *p* > 0.05).

#### Treatment Effectiveness

Participants were also asked to evaluate whether they felt that current treatments for dementia were effective or not. There was no difference between Chinese and DAM participants in the extent to which they felt these were effective (*χ*^2^ (1, N = 135) = .07, *p* = .787). There was also no difference between Chinese people with more or less knowledge of dementia in their beliefs about the effectiveness of current medications using a chi-squared test with Yates’ correction (*χ*^2^ (1, N = 55) = .08, *p* = .773).

#### Willingness to Understand Personal Risk

Participants were asked to indicate their willingness to undertake a range of potential assessments to use different types of diagnostic test or technologies to understand their personal risk of developing dementia (see Table 3). Analysis of these contrasts using Chi-square tests with a corrected significance level of *p* < .008, showed that only one contrast was significant. The Chinese population was significantly more reluctant to use a computer or smartphone to monitor their day-to-day life as a way of informing them about their personal risk of developing dementia (*χ*^2^ (1, N = 198) = 14.01, *p* < .001).

**Table 3.** Participants’ Willingness to Use a Diagnostic Test or Technology to Understand Their Personal Risk

	Chinese UK (*n* = 84)		Dementia attitudes monitor, 3^rd^ wave (*n* = 2,259)	
Willingness of participant to have …	Very or fairly willing (%)	Very or fairly unwilling	Very or fairly willing (%)	Very or fairly unwilling (%)
A memory or thinking test	100	—	94	5
A brain scan	85	14%	91	8
A lumbar puncture or ‘spinal tap’	37	62%	43	54
A blood test	89	11%	94	5
An eye test	92	8%	95	4
A computer or smartphone monitor your day-to-day life	61	39%	83	15

#### Willingness to Identify an Early Diagnosis of Alzheimer’s Disease

Participants were asked to respond to the possibility that they could take a test that could tell them whether they were in the very early stages of Alzheimer’s or another form of dementia, even before symptoms showed. Using a chi-squared test with Yates’ correction suggested a greater preparedness to take a test in the Chinese population (*χ*^2^ (1, N = 197) = 4.58, *p* = .032). However, this was not significant at our adjusted level of .013. There was no difference between Chinese people with more or less knowledge of dementia in their preparedness to take a test (*χ*^2^ (1, N = 84) = .42, *p* = .517).

#### Identifying Personal Risk

Chinese participants were significantly more likely to say that they would want to know information in midlife about their risk of developing dementia in later life than were respondents from the 2023 DAM report (*χ*^2^ (1, N = 198) = 20.14, *p* < .00001). There was no difference in their willingness to know information in midlife between Chinese people with more or less knowledge of dementia.

## Discussion

Dementia care for people from many minority ethnic communities in the UK is marked by a series of health inequalities (Mukadam et al., 2022; Parveen et al., 2017, 2020; Shafiq et al., 2024). This is also likely to be the case for people from Chinese communities (Baghirathan et al., 2020). Chinese people living in the UK, like those from other minority ethnic communities, tend to make less use of health services than do other ethnic minority groups in the UK (Parveen et al., 2020) with older people, in particular, being more likely to be excluded from health services with often limited opportunities or ability to communicate with others outside their communities (Wong et al., 2024; Zhang et al., 2023). This paper reports data from a wider study which has been the first in the UK to attempt to chart knowledge about brain functioning and dementia and to trial an intervention aimed at improving this (Lim et al., 2025). As such our focus on UK Chinese communities is entirely consistent with the “core challenge” of the third Dementia Attitudes Monitor report: namely to engage with underserved audiences, including ethnic minority groups. While this report increased the number of respondents from minority ethnic backgrounds it was still only able to describe data from people who identified as belonging to Asian/Asian British and Black African/Caribbean/ Black British communities. This left people from other community groups (including Chinese communities) either unrepresented or subsumed within the term ‘ethnic minority’.

We set, out, therefore, to replicate elements of the DAM methodology but to focus specifically on the Chinese community living within the UK. We translated the survey into both Traditional and Simplified Chinese and circulated a link to the online survey through Chinese community groups as well as through social media platforms and national and local Chinese networks. In total 84 UK participants (65 women and 19 men) completed the survey, most of whom had either Cantonese or Mandarin as their preferred language and identified China, Hong Kong or Malaysia as their country of origin.

Participants in our survey were more likely to describe themselves as knowing someone who had dementia compared to respondents who identified as being from an ethnic minority in the 2023 DAM report. It is also relevant to note that much of the literature (e.g., Cheston et al., 2017; Wong et al., 2024; Xue et al., 2023) suggests that the Chinese population is reluctant to report dementia in themselves or family members due to cultural stigma. While this difference is most likely to reflect that people with personal experience of dementia were more likely to be motivated to complete the survey, and that disclosure is likely to have been facilitated by the anonymity of an online survey, it might also point towards Chinese people living in the UK growing more accepting of the condition. Certainly, there is some evidence that central aspects of Chinese culture such as the hitherto strong culture of care may have become weakened amongst UK Chinese migrants (Chiu & Yu, 2001).

### Practical Implications

People from ethnic minorities make up 18% of the United Kingdom population^5^ of whom Chinese communities comprise just under half a million individuals. Taken as a whole, the Chinese community in the UK is relatively young – with just 13% aged over 60 compared to the UK average of 20%. However, these figures may well change over the next years as increasing numbers of Hong Kongers migrate to the UK through the British Nationals (Overseas) visa scheme. Over the next years, as the UK Chinese community ages, so it will become increasingly important to find ways to address the dementia needs of this community – from reducing the risk of dementia, to improving understanding of dementia and enhancing care provision. This study, therefore, set out to fill some of the gaps in research and identify how attitudes to dementia may contribute to three different types of need:

(1) *Awareness and understanding.* While Chinese respondents in our survey were just as likely as the wider UK population sampled in the 2023 DAM report to agree that dementia affects both the mental and the physical aspects of a person’s health, there are nevertheless important differences between the two populations. Thus, participants in the 2023 DAM survey were more likely to agree that dementia was an inevitable part of growing older, and that dementia was a cause of death than were Chinese participants in our survey. While greater knowledge of brain functioning as identified by responses to the Think Brain Health Quiz, was associated with viewing dementia as a cause of death, counter-intuitively it was also associated with being more likely to view it as an inevitable part of ageing. It is important, therefore, not only that information campaigns to improve public understanding of brain health be made available in Simplified and Traditional Chinese (and spoken versions in Mandarin and Cantonese), but also that they should be provided in formats and places which Chinese people access.
(2) *Diagnosis and risk.* Chinese participants were just as knowledgeable as those in the DAM survey about whether a range of health conditions were preventable and whether current treatments for dementia were effective. However, participants in our study were significant more likely to say that they would want to know information in mid-life about their risk of developing dementia in later life and were just as willing as were participants in the 2023 DAM study to take a test that could tell them whether they were in the very early stages of Alzheimer’s or another form of dementia, even before symptoms showed. As new forms of dementia modifying treatments such as Lecanemab require early detection, ideally before clinical symptoms become apparent, it is therefore important to ensure that Chinese people have equal access to new medications. Yet, unfortunately, people from many minority communities (Parveen et al., 2020) including Chinese communities (Wong et al., 2024; Zhang et al., 2023) are likely to have less access and at a later stage to many dementia services. Memory Assessment Services, then, will need to proactively engage with Chinese populations if they are to ensure equality of access to new treatments.
(3) *Stigma.* The results from our study are consistent with qualitative reports (Cheston et al., 2017; Wong et al., 2024) pointing to high levels of stigma around dementia within Chinese communities. Thus, Chinese participants were more likely both to say that they would find it hard to talk to someone with dementia and that they would not feel comfortable telling people outside their close family if they were to be diagnosed with dementia. Higher levels of knowledge about dementia were also associated with increased reluctance to tell people outside their family. These findings may be suggestive of a combination of cultural, language and social factors acting as a barrier to prevent Chinese families from engaging effectively with statutory dementia services. Instead, caregivers are more likely to turn to Chinese led, locally based services which they feel will be more likely to understand their needs (Baghirathan et al., 2020). Consequently, as with other minority ethnic communities there is a pressing need for health and social care organisations to find ways to work in partnership with locally based community, faith and voluntary sector groups (Cheston et al., 2025).

#### Strengths and Limitations

This study has been the first to explore attitudes towards dementia and knowledge about brain health within the Chinese population living in the UK – and as such it extends our understanding about a population which has hitherto been almost entirely ignored. Key to our ability to do this was the co-operation of Chinese community organisations, whose support enabled us to access more potential participants than we would otherwise have been able to. Our replication in translated form of an established methodological process enabled us to draw tentative comparisons between the Chinese community and the wider UK population. We were also able to follow the DAM methodology by weighting our data for sex and age, using the recently published data from the 2021 census.

At the same time a number of factors limit our ability to draw substantial conclusions. Importantly, we were only able to recruit 84 participants, and as the DAM authors set out, the smaller the size of the sub-group, then the less we can rely on the survey estimates to be truly representative of the population as a whole. Importantly, our sample size fell below the rule of thumb that they set out whereby groups with fewer than 100 participants could be subject to confidence intervals of up to 10%. Moreover, unlike the three waves of the DAM which drew on either face-to-face (2018 survey) or telephone (2021 and 2023 surveys) interviews, we used an online methodology – which impacted on our ability to frame some questions.

#### Conclusions

Improved public understanding of dementia is a crucial way to reduce dementia risk and to improve quality of life for individuals and their caregivers because it increases awareness, promotes early detection, and empowers individuals to take proactive steps. By understanding dementia, people can learn to recognize symptoms, make informed choices about their health, and access necessary support. Yet, while the UK Chinese community is growing, we know little about the attitudes to dementia of Chinese people living in the UK. While welcome, those recent research studies that have taken place tend to be qualitative in nature and are necessarily quite limited in scope (Bifarin et al., 2022; Truswell et al., 2019). This study has tried to fill in some of the gaps in our knowledge about dementia attitudes amongst the UK Chinese community – for instance participants in our study were highly motivated to reduce their dementia risk. However, if we are to enable Chinese people to make these changes, then this requires specific public health programmes that are targeted on Chinese communities and are adapted to meet their specific needs (Lim, 2024; Xiao et al., 2022). The second stage of this project reports just such a programme (Lim et al., 2025).

## Supplemental Material

Supplemental Material - Attitudes to Brain Health and Dementia Amongst the Chinese Population Living in the UK Supplemental Material for Attitudes to Brain Health and Dementia Amongst the Chinese Population Living in the UK by Richard Cheston, Mei Champ, and Jennifer N. W. Lim in Dementia

## Notes

1. In order to represent the phonetic sounds of Mandarin in this paper, we have used the Pinyin system which is the most widely-used system of writing Mandarin Chinese using the Latin alphabet.

2. Population of England and Wales - GOV.UK Ethnicity facts and figures (ethnicity-facts-figures.service.gov.uk).

3. Application reference: 1221JLUOWHEA (approved on the 11th of March 2022).

4. https://nrs.co.uk/nrs-print/lifestyle-and-classification-data/social-grade/.

5. https://www.ethnicity-facts-figures.service.gov.uk.
