# Supplementary material for: Attitudes to Brain Health and Dementia Amongst the Chinese Population Living in the UK
Source: Dementia (London). 2025 Jun 29;24(8):1632–45. doi: 10.1177/14713012251356529 (PMC12508493; doi:10.1177/14713012251356529)
Supplement: Supplemental Material - Attitudes to Brain Health and Dementia Amongst the Chinese Population Living in the UK [file sj-pdf-1-dem-10.1177_14713012251356529.pdf]

Appendix One: Think Brain Quiz (https://www.alzheimersresearchuk.org/brain-health/take-our-quiz/)

Question 1: *There are more connections between nerve cells in your brain than there are stars in the Milky Way?* (Response options: true, false)

Answer: The brain is the most complex thing in the universe. There are around 100 billion nerve cells (known as neurons) in the brain, each one connected to thousands of other cells.

Question 2: *How much of your energy does your brain use?* (Response options: 5%, 16%, 20%)

Answer: Your brain is only 2% of your total body weight but uses about 20% of your energy. Brain cells use much more energy than other cells in your body and they're always active, even when you're asleep.

Question 3: *Which part of your brain is most responsible for your personality?* (Response options: front of the brain, middle of the brain, back of the brain, sides of the brain)

Answer: The front of the brain (frontal lobe) is responsible for your personality and movement. It's what makes you… you.

Question 4: *True or False? Alzheimer's disease, the most common cause of dementia, can physically shrink the brain?* (Response options: true, false)

Answer: Alzheimer's disease breaks down connections between cells and can shrink the brain by up to 140g. That's about the weight of an orange. But this shows that Alzheimer's is a physical disease and not a normal part of getting older.

Question 5: *How many people are thought to be living with dementia worldwide?* (Response options: 12 million, 38 million, 55 million).

Answer: Nearly 1 million people are living with dementia in the UK and 55 million people worldwide. Sadly, this number is on the rise, with more families facing the devastating impact of dementia every year.

Question 6: *Looking after which of these organs has the greatest impact on dementia risk?* (Response options: your lungs, your kidneys, your heart).

Answer: What's good for your heart is good for your brain. Looking after your heart health, including controlling blood pressure and being physically active, can help lower your risk of heart disease and dementia too – especially Alzheimer's disease and vascular dementia.

Question 7: *As well as looking after your heart health, evidence suggests that staying socially active could help to keep your brain healthy.* (Response options: true, false)

Answer: Evidence suggests that...

- Staying socially active
- Challenging your brain regularly
- Looking after your heart health

...all play a role in helping to protect your brain health.

Question 8: *What percentage of dementia cases are linked to risk factors we may be able to change?* (Response options: 10%, 25%, 40%)

Answer: Our age and genetics play a part in shaping our dementia risk and we can't change these. But research suggests that up to 40% of cases could be linked to risk factors we can influence. Looking after our hearts, regularly challenging our brains and staying connecting to the people around us can help reduce our risk of developing dementia.
